# Investigating the Optimal Membrane‐Based Separation of Cynaroside From Peony Seed Meals and Assessing Its Biomedical Implications

**DOI:** 10.1002/fsn3.4528

**Published:** 2024-12-04

**Authors:** Wen‐Tao Chen, Jing Sun, Ying‐Yang Zhang, Ying Xu, Ping Zou, Jian‐Gang Hu, Lei Zhou

**Affiliations:** ^1^ School of Biological and Food Engineering Changzhou University Changzhou China; ^2^ Department of Gardiology Jintan Affiliated Hospital of Jiangsu University Changzhou China; ^3^ Jiangsu Key Laboratory of Medical Science and Laboratory Medicine, School of Medicine Jiangsu University Zhenjiang China; ^4^ Shaoxing Food and Drug Testing Institute Shaoxing China

**Keywords:** bioactivity, cynaroside, membrane separation, molecular docking, pharmacophore screening, structure–activity relationship

## Abstract

This comprehensive study focused on evaluating and selecting seven distinct commercial membranes to develop BTESE/PA membranes. This method effectively facilitated the extraction of cynaroside from the complex composition of peony seed meal. We subsequently conducted a thorough investigation into its biological properties. These findings establish a robust foundation for future research and the development of related products. The peak concentration achieved by peony seed meal filtration (PSMF) was 234.84 ± 1.17 μg/mL. Among the commercial membranes evaluated, the PA membrane exhibited superior separation capabilities, leading to its selection for BTESE loading. Compared with BTESE treated with HCl and NH_3_, the HCl variant, once incorporated into the BTESE/PA membrane, enhanced cynaroside separation, achieving an impressive 90.23% recovery rate. A comprehensive investigation of the biological effects of cynaroside revealed its crucial antioxidant role, especially in SOD binding. Additionally, cynaroside has the potential to induce apoptosis in K562 cells through interactions with BCL‐2 and CDK‐2 enzymes. Pharmacophore screening revealed the affinity of cynaroside for the PDE5A, TNKS2, and DAPK1 proteins, suggesting that it has diverse potential applications.

## Graphical abstract

Optimize the extraction conditions of flavonoids from peony meals. Cynaroside was isolated and purified by BTESE/PA membrane with a separation rate of 90.23%. The inhibition of K562 cancer cell proliferation by Cynaroside combined with molecular dynamics was discussed. Target protein screening of Cynaroside by pharmacophore method.

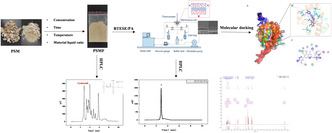

In recent decades, the global recognition of biomass waste as a valuable resource for bioapplications has increased significantly (Erb and Gingrich 2022). The oil peony, a notable species, offers significant economic, ecological, ornamental, medicinal, and oil production benefits (Deng et al. 2022). According to the China Oil Peony Industry Alliance, ~10 million mu of oil peony support numerous peony seed oil companies nationwide. However, the emphasis on peony seed oil production has resulted in the underutilization of byproducts such as seed shells and meal (Bai et al. 2021). The extracted peony seed meal is often discarded or used as fertilizer or animal feed, indicating low utilization. Consequently, developing a refined oil peony processing industry is essential for increasing farmers' income and reducing poverty. Flavonoids, secondary metabolites synthesized during plant photosynthesis, exhibit a wide range of biological activities (Tagde et al. 2021). Its biological functions include anti‐inflammatory and antioxidant effects, enhancement of gastric mucosal regeneration (Wu et al. 2023), prevention of neurodegenerative diseases (Grewal et al. 2021), amelioration of acute respiratory distress syndrome (Rahman et al. 2022), and anticancer effects (El‐Seedi et al. 2021). Apoptosis, a regulated process of self‐destruction, is crucial for maintaining an organism's health and stability (AbouLaila et al. 2020). Flavonoids regulate apoptosis via diverse cellular signaling pathways by either activating or inhibiting caspases, modulating BCL‐2 family proteins, or altering p53 protein expression, all of which are crucial for controlling apoptosis. Through these interactions, flavonoids induce apoptosis in cancer cells, thus inhibiting tumor growth (Kabir et al. 2021). Membrane separation, a physical method, avoids new impurities and chemical reactions, preserving purity (De Rosa et al. 2023). This highly automated, accurate, and consistent process produces colorless, temperature‐stable concentrates with minimal equipment, ensuring a small system footprint. This effectively reduces production costs and investment. Nanofiltration selectively separates organic compounds by adjusting the substrate and coatings to achieve different molecular weights (Saboe et al. 2022). To retain small flavonoid molecules (180–280 Da), a highly dense membrane is needed. Thus, the separation membranes must be extremely dense to accommodate the retention of small flavonoid molecules, and their pore sizes are influenced by the polarity and nonpolarity of flavonoids (Tao et al. 2024).

In this study, an ethanol–water mixture was used to extract byproducts from peony seed oil, and a laboratory‐developed BTESE/PA composite membrane was used to separate the crude extracts. The authors assessed the antioxidant effects and the inhibition of K562 cell proliferation, elucidating the underlying mechanisms from a molecular dynamics perspective. This method facilitates further investigation into the biological properties of cynaroside. The application of high‐purity seed meal flavonoids in pharmaceuticals, healthcare, food processing, and cosmetics can transform research, enhance local agriculture, advance local cultivation, significantly increase farmer incomes, and offer substantial social benefits.

## 1 Materials and Reagents

Oil peony seed meal: China Jiangsu Guose Tiantian Oil Peony Technology Development Co. Ltd.; Rutin, 1,2‐bis(triethoxysilyl)ethane: analytically pure, Aladdin Reagent Co. Ltd.; DPPH, ascorbic acid, o‐phenanthroline, hydrochloric acid, nitroblue tetrazolium chloride (NBT), reduced nicotinamide adenine dinucleotide (NADH), 5‐methyl phenazine methyl sulfate (PMS): analytically pure, Sinopharm Chemical Reagent Co. Ltd.; 1,2‐bis(triethoxysilyl)ethane: analytically pure, Aladdin Reagent Co. Ltd.

**FIGURE 1:**
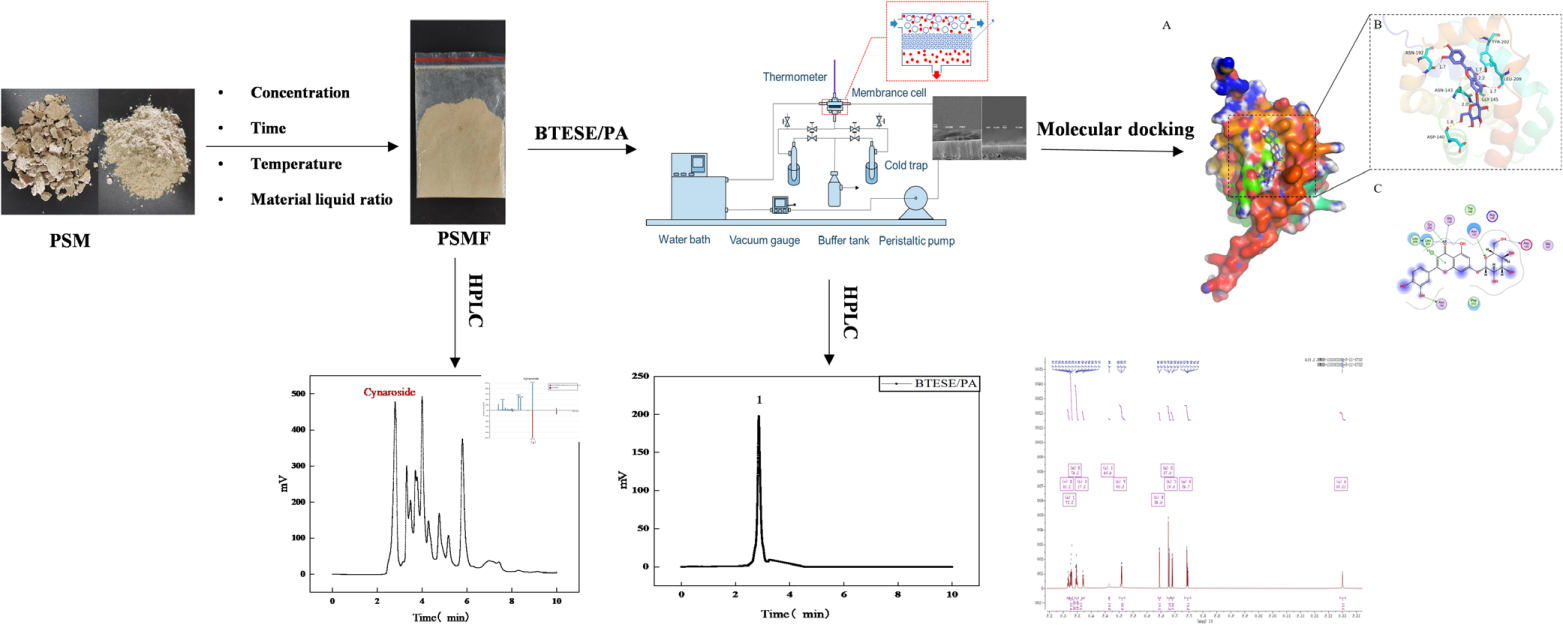
Research concepts related to cynaroside.

The following ultraviolet–visible–near infrared spectrophotometers were used: UV‐3600, Shimadzu Enterprise Management (China) Co. Ltd.; precision electronic balance: ATY224, Changzhou Wantai Balance Instrument Co. Ltd.; high‐throughput tissue disruptor: Tissuelyser‐48, Shanghai Jingxin Industrial Development Co.; mass spectrometer: Q Exactive, Thermo Fisher Scientific; UPLC: Acquity, Waters; vortex mixer: XH‐T, Baita Xinbao Instrument Factory, Jintan District; and nitrogen purge: Reacti‐thermo, Thermo Fisher Scientific (see Figure 1).

## 2 Experimental Methods

### 2.1 PSMF Extraction

**FIGURE 2:**
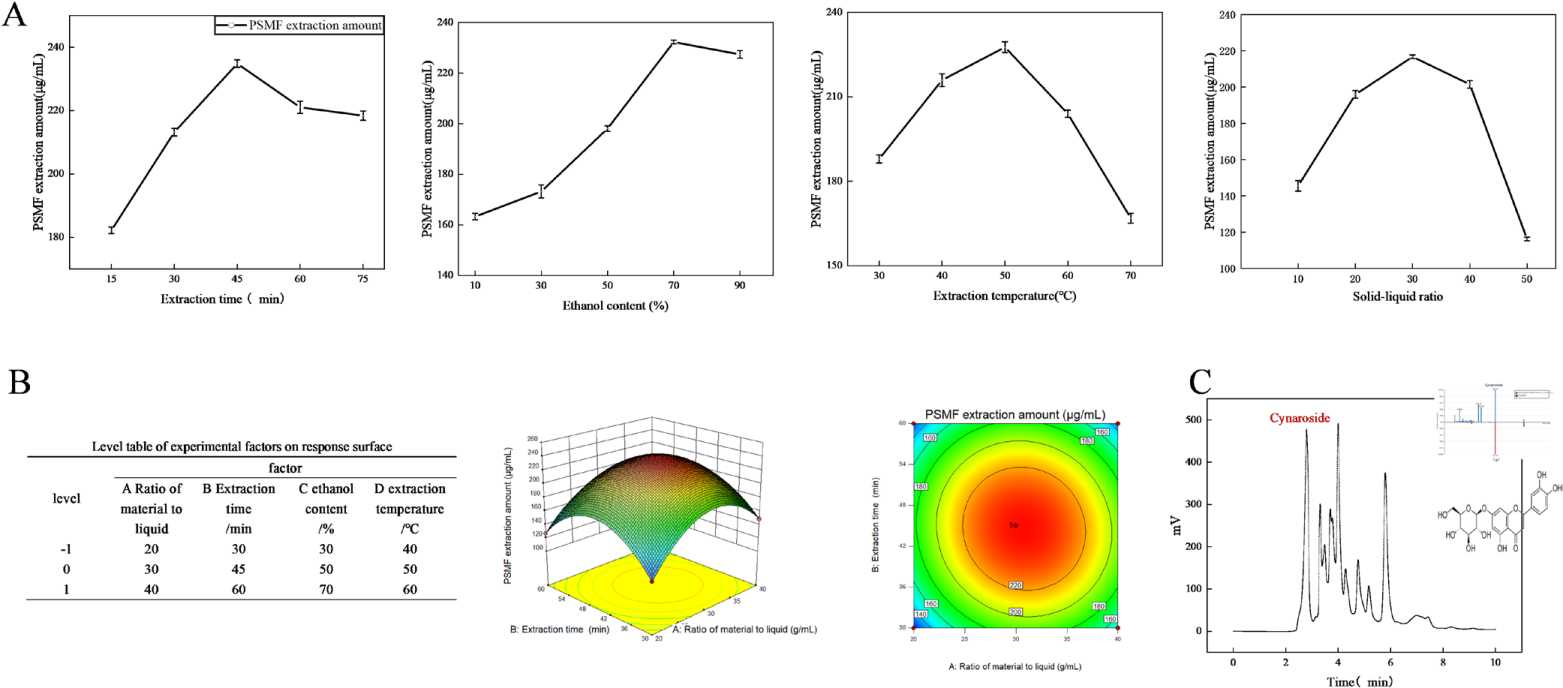
Extraction and identification of cynaroside from PSMF.

Peony seed meal (PSMF) was prepared for experimentation by finely grinding it via a crusher and subsequently sifting it through an 80‐mesh sieve. For the single‐factor experiments, 1.0 g of the prepared PSMF powder was utilized. The experiment involved various conditions, including extraction time (15–75 min), ethanol volume fraction (10%–90%), extraction temperature (30°C–70°C), and solid–liquid ratio (m peony seed meal:V ethanol from 1:10 to 1:50 g/mL). The factor levels for the response surface experiment were determined on the basis of the single‐factor experimental outcomes. The response surface methodology included four primary variables: extraction time, temperature, ethanol concentration, and the liquid–solid ratio. The primary outcome measured was the quantity of extracted flavonoids. This response surface experiment on peony seed meal utilized Design Expert 8.0.6 software. The details and results of this experiment, including specific factor levels and responses, are presented in Figure 2B.

### 2.2 Membrane Screening

Ultrafiltration membranes made of polyvinylidene fluoride (PVDF), polyether sulfone (PES), polytetrafluoroethylene (PTFE), polypropylene (PP), mixed cellulose (MCE), polyacrylonitrile (PAN), and poly and polyamide (PA) were selected for primary separation of the PSMF extract.

The membrane flux (J), water contact angle (WCA) (Hong et al. 2022), and SEM (Meng and Nicolai 2023; Zhang, Wang, et al. 2023; Zhang, Jiang, et al. 2023; Zhang, Li, et al. 2023) of the PSMF‐permeating lyophilized powder were characterized to determine the membrane with the greatest separation effect. Under the conditions of an operating pressure of 0.85 MPa, an operating temperature of 30°C, and a flow rate of 10 mL/min, the membrane flux was measured after the membrane was prepressed for 30 min. The formula for calculating the membrane flux is as follows:

$$J=\frac{W}{T\times A}$$

where *J* represents the membrane flux kg/(m^2^ h), *W* represents the mass of the membrane solution kg, *T* represents the sampling time *H*, and *A* represents the effective area m^2^ of the membrane.

The rejection rate (*R*) (Bargeman 2023) is an important index of membrane separation technology. Owing to different calculation formulas, different values are obtained. Retention is the ability of a membrane to prevent a component in the feed from passing through or retaining one of the other components. The formula for calculating the retention rate is as follows:

$$R=\left(1-\frac{C_{1}}{C_{0}}\right)\times 100\%$$

where *R* represents the percent rejection, *C*_1_ represents the concentration of the permeate, and *C*_0_ represents the concentration of the feed.

### 2.3 Preparation of the BTESE/PA Membrane

The precursor was 1,2‐bis(triethoxysilyl)ethane (BTESE). An organosilica sol was prepared by using 99.7% n‐propanol (NPA) as the solvent. Two kinds of BTESE sols were prepared: acid‐catalyzed (HCl) and base‐catalyzed (NH_3_). The molar ratio of BTESE/H_2_O/HCl (NH_3_) was 1/100/0.15, and the sol solvent was prepared by hydrolysis with water and catalyst at 40°C for 2.5 h. The precursor concentration of both sols was maintained at 5.0 wt%.

Preparation of the BTESE/PA composite membrane: BTESE sol solution was loaded on the surface of the PA membrane via ultrasonic spraying. One of the key aspects of manufacturing homogeneous nanocomposite membranes is ensuring the uniform distribution of nanoparticles in the polymer matrix. The experimental process parameters for membrane preparation were as follows: n‐propanol/water two‐solvent system, 5 wt% BTESE, power of 1.5 W, and gas carrier flow of 0.02 MPa, total pressure 0.6 MPa, spray height 15 mm, flow rate 0.3 mL min^−1^, spray step 2 mm, substrate temperature 25°C.

A certain amount of prepared BTESE sol was injected into the syringe, which was then pushed to clean the pipeline. The program is started, the BTESE sol is pushed into the pipeline by the syringe pump, the ultrasonic controller recognizes the signal, and the BTESE sol is ultrasonically dispersed into tiny uniform droplets and then enters the deposition chamber with the carrier gas (nitrogen). The tiny droplets were uniformly deposited on the PA support from the spray nozzle. After one deposition at the XY fast speeds of 250 and 50 mm s^−1^ and at the shaft speed, the solvent was naturally volatilized for 2 min. The sprayed PA support was sprayed at 120 and dried for 20 min to obtain the BTESE organosilicon composite membrane. A schematic of the BTESE silicone composite membrane prepared via the ultrasonic spraying method is shown in Figure 4A.

### 2.4 Bioactivity Study

This study focused on testing the antioxidant activity and ability to inhibit cancer cell proliferation.

#### 2.4.1 Antioxidant Activity

##### 2.4.1.1 DPPH Radical Scavenging Activity Measurement

Modified slightly from reference (Man et al. 2023). The samples were prepared by combining 2 mL of a cynaroside solution (at concentrations of 50, 100, 150, 200, and 250 μg/mL) with 2 mL of a 0.1 mM DPPH· solution in 95% ethanol. For the blank sample, 2 mL of 95% ethanol was combined with 2 mL of 0.1 mM DPPH· solution. The reaction was carried out in the dark at 37°C for 30 min, and the absorbance was measured at 517 nm.

The scavenging rate calculation formula is as follows:

$$\text{DPPH}\cdot \text{scavenging rate}\;\left(\%\right)=\frac{\left(A_{b}-A_{s}\right)}{A_{b}}\times 100$$

where *A*_s_ and *A*_0_ represent the absorbances of the sample and the blank, respectively.

##### 2.4.1.2 ˙OH Radical Scavenging Activity Measurement

This approach slightly improved upon the method described in reference (Cañas et al. 2023). The mixture included 4 mL each of 1,10‐phenanthroline (5 mM) and FeSO_4_ (5 mM), to which 3 mL of phosphate buffer was added (prepared by mixing 16 mL of 0.2 mol/L sodium dihydrogen phosphate and 84 mL of 0.2 M disodium hydrogen phosphate, pH 7.4), 3 mL of H_2_O_2_ (0.01%), and 4 mL of cynaroside solution (50, 100, 150, 200, 250 μg/mL), and the mixture was stirred and left to stand for 1 h. The absorbance was measured at 536 nm. The PSMF solution was replaced with distilled water for the control group and with hydrogen peroxide for the blank group.

˙OH scavenging rate formula:

$$˙\mathrm{OH}\;\text{scavenging rate}\;\left(\%\right)=\frac{A_{s}-A_{c}}{A_{b}-A_{c}}\times 100\%$$

where *A*_s_, *A*_c_, and *A*_b_ represent the absorbances of the sample, control, and blank, respectively.

##### 2.4.1.3 ˙O_2_ ^−^ Radical Scavenging Activity Measurement

Adapted from reference (Martinelli et al. 2021), 1.5 mL of cynaroside solution (50, 100, 150, 200, 250 μg/mL) was mixed in sequence with 0.5 mL of 0.30 mM NBT (prepared in pH 8.0 Tris–HCl), 0.5 mL of 0.468 mM NADH (prepared in pH 8.0 Tris–HCl), and 0.5 mL of 0.060 mM PMS (prepared in pH 8.0 Tris–HCl), mixed well and then incubated in a 25°C water bath for 5 min. The absorbance was measured at 560 nm.

The superoxide anion scavenging rate formula was as follows:

$${˙O}_{2}^{-}\;\text{scavenging rate}\left(\%\right)=\frac{1-A_{s}}{A_{0}}\times 100\%$$

where *A*_s_ and *A*_b_ represent the absorbances of the sample and blank, respectively.

##### 2.4.1.4 MTT Assay for PC12 Cell Viability

Using the method of Niemietz and Brown (2023), we precultured logarithmically growing PC12 cells for 24 h. After removing the medium from the wells, 100 μL of serum‐free medium containing 0, 50, 100, 150, 200, or 250 μM cynaroside was added, with a control group and a blank group set up, each with four replicates. After 24 h of treatment, 10 μL of MTT solution was added to each well and incubated for 4 h. The supernatant was discarded, and 100 μL of formazan solvent was added to all the wells, mixed, and incubated for approximately 4 h until no purple crystals were observed under an inverted microscope. The absorbance of each well at 570 nm was measured via an enzyme marker.

The survival rate was calculated as follows:

$$\text{Cell survival rate}\left(\%\right)=\frac{A_{s}-A_{b}}{A_{c}-A_{b}}\times 100\%$$

where *As*, *A*_b_, and *A*_c_ represent the OD of the experimental group, blank group, and control group, respectively.

##### 2.4.1.5 Intracellular ROS Content Measurement

In accordance with the methods of Xiong et al. (2021), PC12 cells were seeded at a density of 1 × 10^5^ cells/mL in sterile all‐black 96‐well plates. Once the cells reached approximately 80% confluence, 100 μL of serum‐free medium containing 50, 100, 150, 200, or 250 μM cynaroside was added. After 24 h, the old medium was discarded, and 100 μL of a 10 μM fluorescent probe was added and incubated for 20 min. After incubation, the cells were washed 1–2 times with PBS to remove probes that did not enter the cells. Finally, 100 μL of PBS buffer was added to each well, and the fluorescence intensity was measured in the dark with a fluorescence enzyme marker at excitation and emission wavelengths of 495 and 529 nm, respectively.

The intracellular ROS levels after BPH treatment were calculated via the following formula:

$$\mathrm{ROS}\left(\%\right)=\frac{A_{s}-A_{b}}{A_{c}-A_{b}}\times 100\%$$

where *A*_s_, *A*_b_, and *A*_c_ represent the OD of the experimental group, blank group, and control group, respectively.

##### 2.4.1.6 Intracellular Antioxidant Activity (CAA) Measurement

Logarithmically growing PC12 cells were adjusted to a density of 6 × 10^4^ cells/mL and seeded in sterile all‐black 96‐well plates, after cell culture, the old medium was removed, and the cells were washed 1–2 times with sterile D‐Hank's solution to remove cells that were not fully adherent or dead. The experimental group was supplemented with 100 μL of serum‐free medium containing various concentrations of GSH and DCFH‐DA probes (25 μmol/L), whereas the control group and blank group were supplemented with 100 μL of serum‐free medium supplemented with DCFH‐DA probes. The cells were then incubated at 37°C in a 5% CO_2_ incubator for 1 h and washed 2–3 times with sterile D‐Hank's solution, after which 100 mL of ABAP solution (600 μM) was added to the experimental and control groups, whereas 100 μL of sterile D‐Hank's solution was added to the blank group. Immediately, fluorescence was measured in real time with a fluorescence enzyme marker at 5 min intervals and for 1 h at an excitation wavelength of 485 nm and an emission wavelength of 538 nm.

The CAA value calculation formula is as follows:

$$\mathrm{CAA}=\left(1-\frac{S_{A}}{C_{A}}\right)\times 100\%$$

where *S*_A_ is the area under the curve of the fluorescence value over time after adding Cynaroside, and *C*_A_ is the area under the curve of the fluorescence value over time for the blank group.

##### 2.4.1.7 Molecular Docking

The antioxidant enzymes used for molecular docking included SOD (PDB ID: 1PM9), CAT (PDB ID: 3QJ4), POD (PDB ID: 1M9Q), and GPX (PDB ID: 6ELW). Protein structures were processed via AutoDock Tools (v1.5.6) for hydrogen addition, charge calculations, and atom type assignments and saved in “pdbqt” format. Crystalline water and the raw ligands were removed via PyMOL 2.3.0. The binding sites were predicted with POCA 1.1, and docking was performed via AutoDock Vina 1.1.2. The parameters for SOD were as follows: center‐*x* = 16.3, center‐*y* = 28.4, and center‐*z* = 74.9, with a search space of 50 × 50 × 50 (grid spacing: 0.375 Å) and an exhaustiveness of 0.375 Å; exhaustiveness: 10; and the remaining parameters were set to their defaults.

#### 2.4.2 Cancer Cell Inhibition Capability

##### 2.4.2.1 Cytotoxicity Assay

Cynaroside solutions at concentrations of 125, 250, 500, and 1000 μg/mL were prepared. Various cell lines (SH‐SY5Y, K562, 293 T, and MCF‐7/ADR cells from the Shanghai Institute of Biochemistry and Cell Biology, China) were maintained in a 5% CO_2_ environment. The cells were seeded at a density of 3 × 10^5^ cells/well in a 96‐well plate and incubated overnight. Different concentrations of cynaroside were added to the cells, which were then cultured for 24 h. The cells were treated with MTT (10 μL, 5 mg/mL) for 4 h, treated with 10% SDS‐HCl (100 μL), incubated at 37°C overnight or washed, and treated with DMSO (100 μL). The absorbances at 570 and 630 nm were measured.

##### 2.4.2.2 Effects of Cynaroside on K562 Cells

Cynaroside was added at concentrations of 0, 62.5, 125, and 250 μg/mL, followed by washing with PBS for 48 h. After 15 min, the cells were stained with DAPI and Annexin V‐FITC/PI in the dark, and the cell cycle distribution and degree of apoptosis were analyzed via flow cytometry.

##### 2.4.2.3 Hoechst 33258 Staining

K562 cells were seeded in a 24‐well plate, cynaroside (0, 62.5, 125, or 250 μg/mL) was added, and the mixture was incubated for 48 h. The cells were stained with Hoechst 33258 (1 μg/mL) for 15 min, washed with PBS, and observed with a fluorescence microscope.

##### 2.4.2.4 Western Blot Analysis

K562 cells were seeded in a 6‐well plate and treated with different concentrations of cynaroside (0, 62.5, 125, or 250 μg/mL). Proteins were separated via SDS–PAGE and transferred onto a PVDF membrane. The cells were incubated with primary antibody for 4 h, washed, and then incubated with secondary antibody for 1 h. After washing, the proteins were visualized via an enhanced chemiluminescence (ECL) detection reagent.

##### 2.4.2.5 Measurement of the Mitochondrial Membrane Potential

K562 cells were seeded in a 6‐well plate, cynaroside was added, and the cells were washed with PBS after 24 h. Rhodamine 123 (5 μg/mL) was added, followed by incubation at 37°C for 30 min. After washing with PBS, flow cytometry was performed for analysis.

##### 2.4.2.6 Research on the Antioxidant Mechanism

BAX, BCL‐2, and CDK‐2 enzymes were acquired from Aladdin Biological Reagent Co. Shanghai, China. Cynaroside was added at concentrations of 25, 50, 100, 150, and 200 μg/mL to measure the ultraviolet absorption spectrum from 190 to 340 nm.

Droplets of the enzymes BAX, BCL‐2, and CDK‐2 with varying concentrations of cynaroside were applied to KBr tablets for data collection, and the ultraviolet absorption spectrum between 1600 and 1700 cm^−1^ was analyzed.

#### 2.4.3 Molecular Dynamics

The crystal structures of the POD, SOD, CAT, and GPX proteins used for docking were obtained from the PDB (Burley et al. 2023). The PDB IDs of the crystal structures were 1M9Q, 1PM9, 3QJ4, and 6ELW. The crystal structures of BAX, BCL‐2 and CDK‐2 were obtained from the Alphafold Database (Mirabello et al. 2024) (https://alphafold.com/entry/A0A7L2ZRC1). The structure of the small molecule cynaroside was obtained from ChemDraw. The database was downloaded, and the energy minimization was performed under the MMFF94 force field (Kim et al. 2016).

AutoDock Vina 1.1.2 software was used in this study. The receptor protein is processed to remove water molecules, salt ions, and small molecules. The docking box was subsequently set up with the center of the box being the center of mass of the ligand in the original crystal, and the box size was 25 × 25 × 25^3^ Å. In addition, ADFRsuite 1.0 was used to convert all processed small molecules and receptor proteins to the PDBQT format necessary for AutoDock Vina 1.1.2 docking. When docking, the detail of the global search was set to 32, and the rest of the parameters remained at the default settings. The docking conformations with the highest scores were the binding conformations for subsequent molecular dynamics simulations and visual analysis of the PyMOL 2.5.4 docking results. With the small molecule cynaroside‐BAX, BCL‐2, and CDK‐2 protein complexes obtained via the above docking method as the initial structures, all‐atom molecular dynamics simulations were carried out via AMBER 18 software. Before the simulation, the charge of the small molecule was calculated via the Hartree–Fock (HF) SCF/6‐31G* of the antechamber module and Gaussian 09 software. After that, the GAFF2 small‐molecule force field and the ff14SB protein force field were used for small molecules and proteins, respectively. Make a description. For each system, the LEaP module was used to add hydrogen atoms to the system, a truncated octahedral TIP3P solvent box was added 10 Å from the system, Na^+^/Cl^−^ was added to the system to balance the charge of the system, and finally, the topology and parameter files for simulation were output.

Molecular dynamics simulations were performed via AMBER 18 software. Before the simulation, the energy of the system was optimized, including 2500 steps of the steepest descent method and 2500 steps of the conjugate gradient method. After the energy optimization of the system was complete, the temperature of the system was slowly increased from 0 to 298.15 K by heating the system for 200 ps at a fixed volume and a constant heating rate. Under the condition that the system is maintained at 298.15 K, a 500 PS NVT (isothermal and isobaric) system simulation is carried out so that the solvent molecules are further uniformly distributed in the solvent box. Finally, in the case of NPT (isothermal and isobaric), 500 ps equilibrium simulations were performed for the entire system. Finally, the two composite systems were simulated by a 50 ns NPT (isothermal and isobaric) system under periodic boundary conditions. The particle mesh Ewald (PME) method was used to calculate the long‐range electrostatic interactions in the simulations with the nonbonded cutoff distance set to 10 Å. The SHAKE method was used to limit the bond length of hydrogen atoms, and the Langevin algorithm was used for temperature control, where the collision frequency *γ* was set to 2 ps^−1^. The system pressure is 1 ATM, the integration step is 2 fs, and the trajectories are saved every 10 ps for subsequent analysis. The binding free energies between the protein and ligand for all the systems were calculated via the MM/GBSA method. In this study, MD trajectories of 45–50 ns are used for calculation, and the specific formula is as follows:

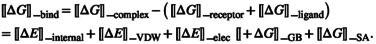




Where 〖Δ*E*〗_internal denotes the internal energy, 〖Δ*E*〗_VDW denotes the van der Waals interaction, and 〖Δ*E*〗_elec denotes the electrostatic interaction. The internal energy includes the bond energy (Ebond), angle energy (Eangle), and torsion energy (Etorsion); [Δ*G*]__GB_ and [Δ*G*]__GA_ are collectively referred to as solvation‐free energy. where [Δ*G*]__GB_ is the polar solvation‐free energy and [Δ*G*]__GA_ is the nonpolar solvation‐free energy. For [Δ*G*]__GB_, this paper uses Nguyen, Roe, and Simmerling (2013) Wait for the GB model developed by the researcher for calculations (igb = 8). The nonpolar solvation‐free energy ([Δ*G*]__SA_) was calculated on the basis of the product of surface tension (*γ*) and the solvent‐accessible surface area (SA), [Δ*G*]__SA_ = 0.0072 × SASA (Weiser, Shenkin, and Still 1999). Entropy changes are ignored in this study because of high computational resource consumption and low accuracy.

### 2.5 Target Screening

To identify the potential proteins of the target molecules, we screened the target compounds with a pharmacophore library. The short peptide molecules were constructed via the Maestro module of the Schrodinger2021‐1 software package. The pharmacophore library was selected from the library reported by Moumbock (Moumbock et al. 2021). The library is based on the latest PDB cocrystal molecules and contains 16,646 pharmacophore models. The potential proteins affected by the target compound can be fully explored.

The phase module of the Schrodinger software package (Dixon, Smondyrev, and Rao 2006) was used for target screening in this study. Considering that there are many rotatable bonds, in the screening process, we set the number of conformations generated for the target molecule to 200 so that we can explore the conformational space of the target molecule as much as possible and define that when all the pharmacophores of the protein are bound, they can be regarded as hit proteins. After screening, the target proteins were further filtered on the basis of the screening score fitness and manual selection.

### 2.6 Data Processing

GraphPad Prism 5 software (GraphPad Software; San Diego, CA, USA) was used for the analysis of the correlations between different variables.

## 3 Results and Discussion

### 3.1 PSMF Extraction

Several critical parameters, including the solid‐to‐liquid ratio, extraction time, ethanol concentration, and extraction temperature, significantly influence the quantitative analysis of the PSMF content (Liu et al. 2023). Individual experiments methodically investigated these variables to delineate their interdependencies. The linear detection range for PSMF ranged from 0.00 to 500.00 μg/mL. This relationship was mathematically expressed as *Y =* (0.03 ± 2.01E‐4) *X* + (0.01 ± 0.006), *R* ^2^ = 0.999 5.

As shown in Figure 2A, the solubility of PSMF in ethanol was a noteworthy aspect of these analyses. A limited solvent volume results in rapid saturation and a reduced yield (Cheng et al. 2024), whereas excessive solvent complicates the concentration process. The optimal solid‐to‐liquid ratio was established at 1:30. The optimal extraction duration was 45 min, yielding a peak of 245.84 ± 1.17 μg/mL, with reduced efficiency observed beyond this period. The yield reduction was attributed to the degradation of the phenolic hydroxyl groups of the flavonoids into less desirable compounds over time. A positive correlation was noted between the increased ethanol volume fraction and the quantity of extractable flavonoids. Excessive ethanol, however, introduces organic impurities, compromising the purity of the extraction process. As a result, a 70% (ethanol/water, V/V) mixture was determined to be the optimal extraction solvent. Temperature control is essential (Chen et al. 2022); appropriate heating activates hydroxyl radicals in flavonoids, whereas excessive temperatures cause oxidation and degradation, especially above 50°C.

The optimal conditions determined from these experiments included a 1:30 solid–liquid ratio, 50°C extraction temperature, 70% ethanol concentration, and 45 min of extraction, yielding a PSMF concentration of 245.84 ± 1.17 μg/mL. Using Design Expert software, the parameters were refined to predict an optimal PSMF concentration of 247.28 ± 2.25 μg/mL (Figure 2B). A mere 0.005% discrepancy between the theoretical and actual extraction processes suggested that the extraction conditions were effectively optimized.

Compound analysis was conducted via a Thermo Ultra‐High‐Performance Liquid Chromatography system (Vanquish, USA) with a Waters HSS T3 column (100 × 2.1 mm, 1.8 μm). The injection volume was maintained at 2 mL, and the column temperature was 40°C. The chromatographic profile comprised two mobile phases: phase A with 0.1% formic acid in acetonitrile and phase B with 0.1% formic acid in water. Chromatographic analysis (Figure 2C) revealed 14 distinct flavonoid compounds, notably, cynaroside (C_21_H_20_O_11_), which constituted 27.89% of the peony seed meal and exhibited a spectral peak at 136 s with a m/z of 449.1.

### 3.2 Membrane Screening

**FIGURE 3:**
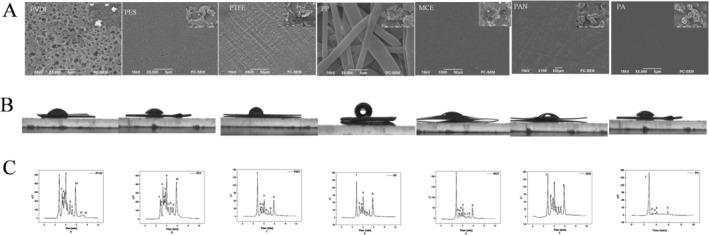
Membrane screening.

Figure 3A shows a detailed comparative analysis of surface morphologies across various ultrafiltration membranes: PVDF, PTFE, PES, PP, MCE, PAN, and PA. Field emission scanning electron microscopy (SEM) with a Zeiss SUPRA 55 was used to obtain diverse morphological characteristics, which is directly attributable to the varied fabrication methodologies applied to these films. Nonwoven fabric PA membranes are distinguished by their exceptional intermolecular cohesion (Zhan et al. 2023). This cohesion is likely due to strong hydrogen bonding from amide groups and complementary proton‐donating species in the target compounds. The SEM imagery vividly portrays the PA membrane surface, emphasizing the uniformity and smoothness of the PSMF freeze‐dried powder particles, which is indicative of a highly consistent ultrafiltration process.

Figure 3B shows the water contact angle measurements of the membranes. The PP membrane, which has the highest contact angle, has a lower hydrophilicity than its counterparts do, whose angles all fall below the 90° mark, indicating hydrophilic tendencies. The PA membrane, with a water flux of 1.98 kg m^2^ h^−1^, is highly hydrophilic, which correlates with its high permeability and retention efficiency. In contrast, the PP membrane shows significantly lower hydrophilicity, as indicated by its minimal water flux.

Figure 3C shows the separation performance of the membranes in processing PSMF, establishing a clear hierarchy of effectiveness: PA > PAN > MCE > PVDF > PES > PTFE > PP. The PA membrane demonstrates superior performance, achieving a flux of 1.98 kg m^2^ h^−1^ and affecting the separation of five flavonoid compounds from the PSMF feed. In comparison, the remaining membranes demonstrated varying degrees of retention, with 6–10 substances being captured, suggesting less separation efficiency. These results strongly support the use of PA membranes as the preferred material for developing advanced composite polyamide nanofiltration membranes.

### 3.3 Preparation of the BTESE/PA Membrane

Membrane technology refinement in materials science incorporates various methodologies to increase membrane functionality (Zhou, Wang, et al. 2023; Zhou, Qu, et al. 2023), durability, and versatility. Key strategies include additive, surface, structural, interface, and synthetic modifications. Additive modification involves incorporating additives such as plasticizers, stabilizers, and antioxidants into the base membrane material to increase its flexibility, environmental tolerance, and degradation resistance. Surface modification involves physical or chemical treatments of the membrane's exterior (Farahbakhsh et al. 2023), such as coating, ion implantation, and chemical grafting, to improve characteristics such as adhesion, anti‐fouling, and smoothness. Structural modifications, adjusting the intrinsic structure of the membrane, alter the pore size, layer thickness, and porosity to fine‐tune the permeability and selectivity. Interface modification, which enhances separation efficiency and specificity, changes the adhesion between the membrane and substrate or modifies its affinity for target molecules. Excessive gel layer deposition may lead to a nonuniform and coarse surface texture.

**FIGURE 4:**
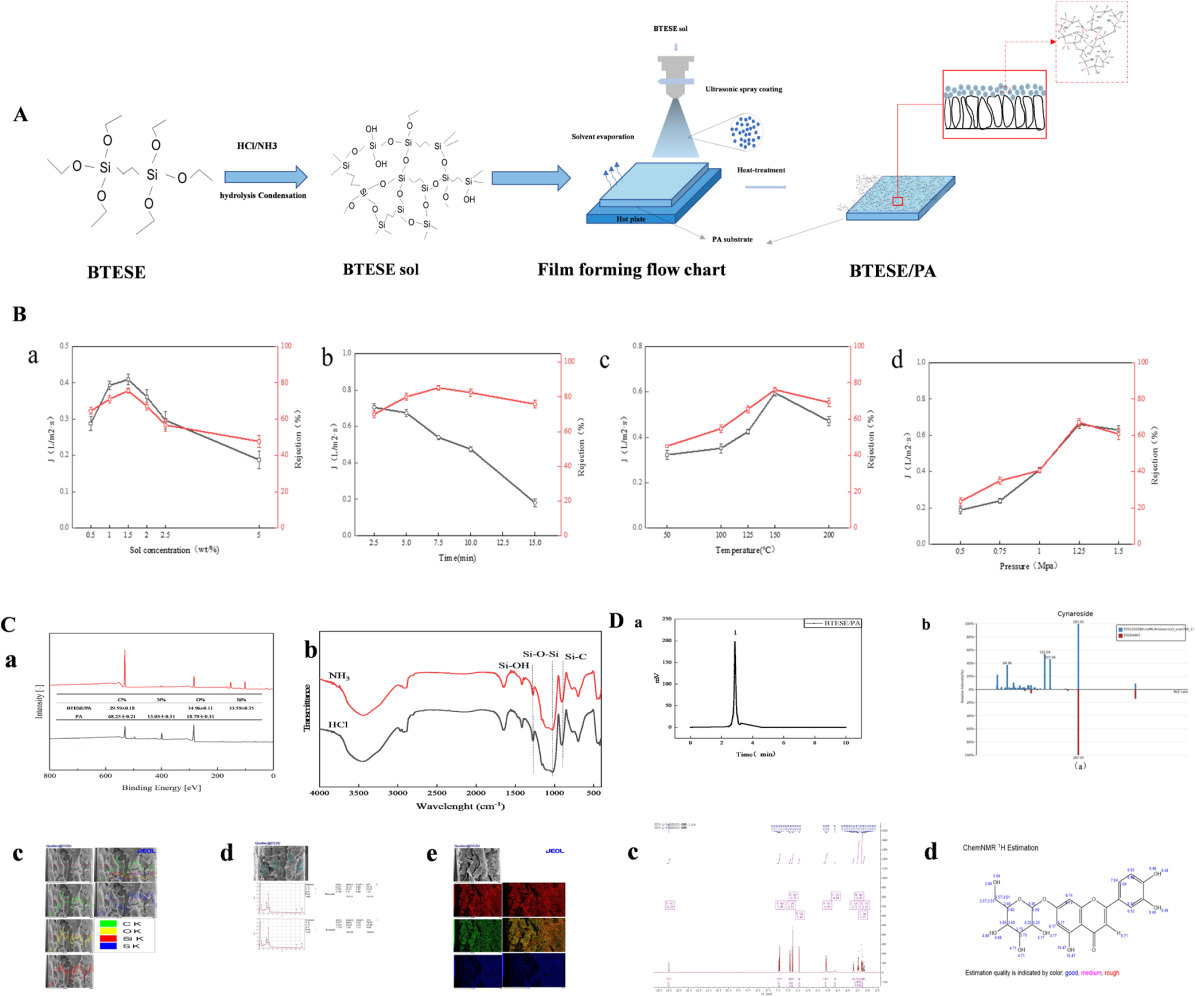
Preparation of the BTESE/PA membrane.

One of the key aspects of manufacturing homogeneous composite membranes is ensuring a uniform distribution of nanoparticles in the polymer matrix. A diagram of the prepared BTESE/PA composite membrane is shown in Figure 4A. The polymer PA carrier was cut into regular quadrangles and soaked in distilled water for at least 12 h to remove glycerin and impurities from the carrier surface layer. The soaked PA film was fixed on a glass plate, and the BTESE sol particles were evenly sprayed on the surface of the PA film via ultrasonic atomization spraying and then heat‐cured in a vacuum drying oven at 100°C for 10 min.

Figure 4B(a) illustrates the exploration of sol concentrations ranging from 0.5 wt% to 5 wt%, using ultrasonic atomization to identify the minimum effective concentration. An optimal separation efficiency, marked by a uniform droplet distribution and stable flow, was attained at a 1.5 wt% concentration. This efficiency results from larger sol particle sizes and greater liquid inlet velocities, leading to the selection of 1.5 wt% BTESE for further investigation. Precise control of the deposition duration and thermal treatment is crucial for ensuring the uniformity and integrity of the resulting separation layer. An inadequate deposition time can lead to incomplete coverage, undermining the effectiveness of heat treatment. Insufficient thermal treatment may result in an overly thin BTESE layer or a failure to form a cohesive film, negatively impacting the film quality and separation capacity, as explained previously. In Figure 4B(b), the influence of heat treatment time on the separation performance of the BTESE/PA composite membrane is examined. Five minutes of thermal exposure produced optimal results, highlighting the need to avoid prolonged heat treatment, which could cause increased interfacial stress and potential cracking due to an overly thickened separation layer. Figure 4B(c) shows the effect of the thermal treatment temperature on the functionality of the BTESE silicone composite membrane. Treatment at 150°C resulted in decreased flux, likely due to shrinkage of the holes in the reducing capacity of the PA support. A higher retention rate at this temperature indicates a change in the properties of the PA support. Figure 4B(d) shows that at 1.25 MPa, there is no significant improvement in the retention rate, indicating that pressure has a limited impact on efficiency, likely because the compression of the PA support under high pressure does not enhance retention. During membrane fabrication, the sol concentration is critical‐low concentrations may not form continuous films, whereas high concentrations can cause cracks. An optimal 1.5 wt% concentration balances droplet uniformity and stability.

Fourier transform infrared spectroscopy (FTIR) analysis, as shown in Figure 4C, revealed Si‐OH and Si‐O‐Si vibrations, confirming successful sol–gel reactions. X‐ray photoelectron spectroscopy (XPS) revealed a change in the surface composition of the BTESE/PA composite film, with increased Si and decreased C and N contents compared with those of the PA substrate. Energy dispersive spectroscopy (EDS, Figure 4C(c–e)) further confirmed the uniform distribution of C, O, and Si, indicating effective BTESE incorporation.

Figure 4D(a,b) shows the efficacy of the BTESE/PA membrane in analyzing the filtrate composition, which aligns with the results of the NMR simulations (Figure 4D(c,d)) and highlights the efficient separation of cynaroside at a concentration of 158.40 ± 2.23 μg/mL and 90.23% recovery.

### 3.4 Biological Activity

Flavonoids, key plant secondary metabolites, play critical roles in various biological processes and plant adaptation to environmental challenges (Zhang, Wang, et al. 2023; Zhang, Jiang, et al. 2023; Zhang, Li, et al. 2023). Flavonoids, which are common in the human diet, are renowned for their antioxidant properties and diverse biological activities, including bacteriostatic effects and cancer cell proliferation inhibition, potentially reducing disease risk. The biological efficacy of flavonoids is influenced by the substitution patterns in their C6‐C3‐C6 ring structures (Zhang, Wang, et al. 2023; Zhang, Jiang, et al. 2023; Zhang, Li, et al. 2023). Despite their importance, comprehensive research on the distribution, biosynthesis, and health benefits of plant flavonoids is still lacking.

**FIGURE 5:**
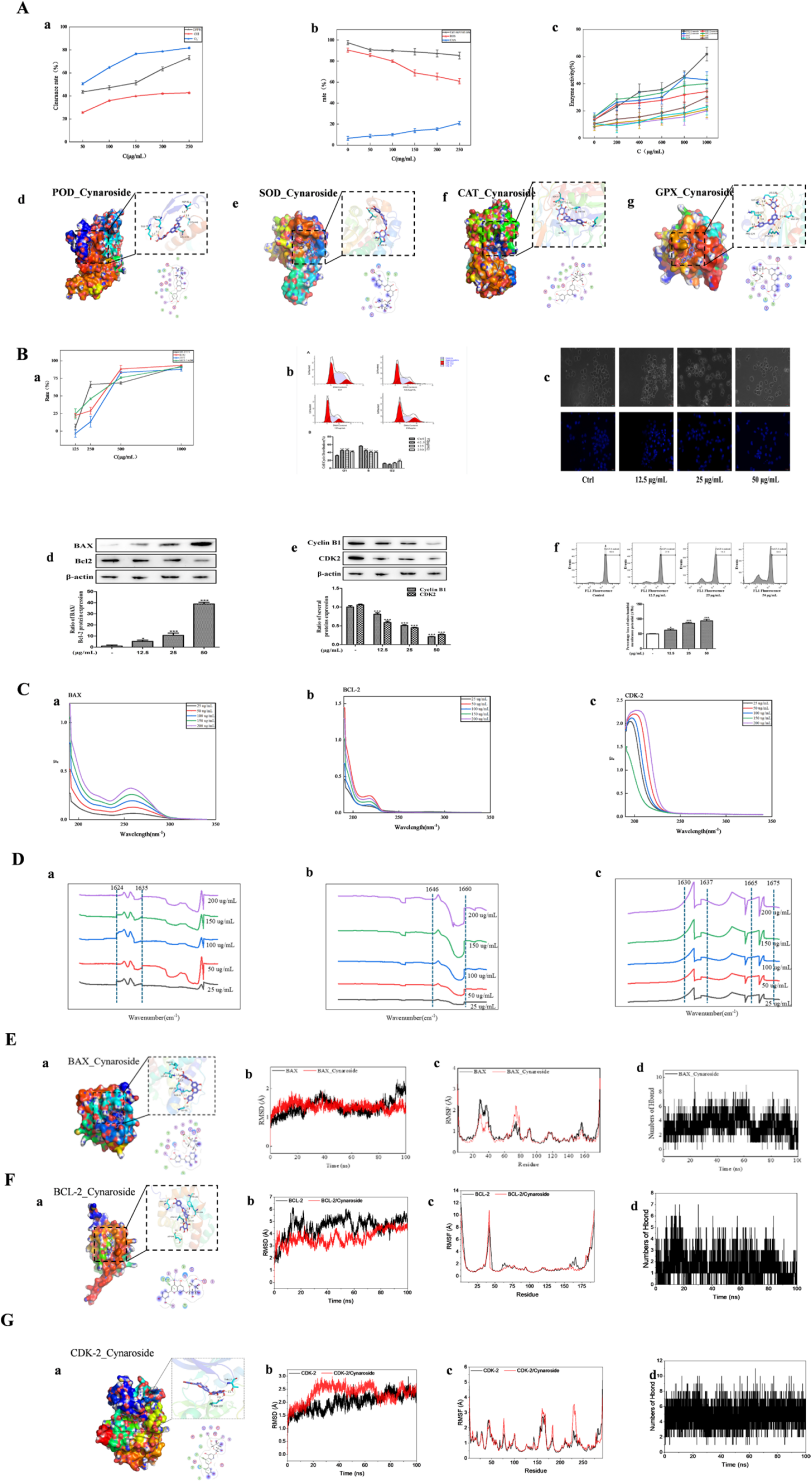
Biological activity of cynecroside (A‐ antioxidant capacity; B‐ inhibited the proliferation of K562 cells; C‐UV–vis; D‐FT‐IR; E‐ BAX‐cynecroside; F‐ BCL‐2‐cynecroside; G‐ CDK‐2‐cynroside).

Cynaroside, a flavonoid derivative, was isolated via a BTESE/PA membrane complex and was carefully prepared through PA membrane modification. This study rigorously tested the antioxidant, antibacterial, and anticancer effects of cynaroside on cell proliferation, highlighting its broad biological efficacy. The antioxidant capacity of cynaroside is significantly influenced by its phenolic hydroxyl configuration. Its hydroxyl (OH) groups increase the binding efficiency and interactions with the polar regions of lipid bilayer membranes (Zhou, Wang, et al. 2023; Zhou, Qu, et al. 2023). Additionally, these hydroxyl groups protect the hydrophobic core of the membrane from alteration. At the lipid–water interface, they serve as a barrier against free radical infiltration, enhancing intracellular antioxidant activity. Figure 5A shows the dose‐dependent increase in the antioxidant potential of cynaroside, which significantly scavenges DPPH, ·OH, and ·O_2_^−^ radicals. Intracellular assays indicated that cynaroside reduces reactive oxygen species (ROS) levels by increasing cellular antioxidant activity (CAA). Flavonoids are recognized for their ability to neutralize free radicals by modulating antioxidant enzymes such as POD, SOD, CAT, and GPX, which are the primary defenses against oxidative stress. Molecular docking studies revealed that cynaroside formed hydrogen bonds with key amino acids in the active sites of the enzymes, confirming its strong affinity. Docking simulation binding affinity calculations support the potential of cynaroside to interact with these enzymes, as further evidenced by increased enzyme activity, especially that of POD, at higher concentrations.

The limited research on the anticancer properties of cynaroside prompted this study, which revealed significant antiproliferative effects on four cancer cell lines, particularly K562 cells. cells. Consequently, K562 cells were chosen for additional cellular pathway analysis. Figure 5B(b) shows that cynaroside induces G1 phase arrest in K562 cells, suggesting that it inhibits cell proliferation by disrupting cell cycle progression (Nagata 2018). Microscopic analysis confirmed that cynaroside induces apoptosis, as evidenced by changes in cell and nuclear morphology. Apoptosis involves mitochondrial pathways; Figure 5B(f) suggests that the cynaroside‐induced apoptosis of K562 cells is correlated with decreased mitochondrial membrane potential and early‐stage apoptosis.

In cancer research, the dynamic equilibrium between the BAX and BCL‐2 proteins is crucial for regulating apoptosis, determining whether cells survive or die (Rajput et al. 2021). Figure 5C shows that the addition of cynaroside glycosides to the BAX, BCL‐2, and CDK‐2 proteins caused an ultraviolet blueshift. According to Figure 5D, adding cynaroside to BAX, BCL‐2, and CDK‐2 induces tensile vibrations under FTIR. The slight blueshift in the amide I band (1600–1700 cm^−1^) suggested hydrophobic and electrostatic interactions. The complexation of proteins with cynaroside enhances the β‐trans structure and improves protein function via electrostatic interactions (Zhang, Wang, et al. 2023; Zhang, Jiang, et al. 2023; Zhang, Li, et al. 2023). Figure 5E,F,G shows that RMSD values from molecular dynamics simulations reveal protein complex movements: higher RMSD and fluctuations indicate more vigorous motion, whereas lower values suggest greater stability. Figure 5F(b) reveals that the BCL‐2‐cynecroside complex stabilized at 20 ns, resulting in a lower RMSD than that of its unbound state, indicating that cynaroside enhances protein stability through interactions. The RMSF quantifies changes in protein flexibility during simulations and is essential for analyzing drug–protein interactions. After drug binding, proteins typically exhibit altered flexibility, which is crucial for modulating enzymatic activity. Figure 5E(c) shows that the BAX‐cynecroside complex has a lower RMSF across its amino acid sequence than unbound BAX does, suggesting that cynaroside stabilizes the protein, potentially impacting its function. Binding energies determined by the MM‐GBSA method offer precise insights into the effects of cynaroside on target proteins. According to Table 1, the binding energies (Δ*G*__bind_) for BAX‐cynecroside, BCL‐2‐cynecroside, and CDK‐2‐cynecroside were −29.00 ± 0.93, −21.65 ± 1.89, and −29.24 ± 1.53 kcal/mol, respectively, with lower values indicating stronger binding affinities. Our findings indicate that these complexes are likely to form, driven mainly by van der Waals (Δ*E*__vdw_) and electrostatic energies (Δ*E*__elec_), with additional contributions from nonpolar solvation free energy (Δ*G*__GB_).

**TABLE 1:** Binding free energies and energy components predicted by MM/GBSA (kcal/mol).

System name	BAX‐Cynaroside	BCL‐2‐Cynaroside	CDK‐2‐Cynaroside
Δ*E*__vdw_	−26.60 ± 0.89	−24.25 ± 1.98	−39.85 ± 2.22
Δ*E*__elec_	−67.70 ± 2.15	−30.20 ± 6.75	−55.13 ± 4.27
Δ*G*__GB_	70.20 ± 2.02	36.85 ± 5.95	72.02 ± 2.58
Δ*G*__SA_	−4.90 ± 0.06	−4.05 ± 0.35	−6.28 ± 0.13
Δ*G*__bind_	−29.00 ± 0.93	−21.65 ± 1.89	−29.24 ± 1.53

Abbreviations: Δ*E*__elec_, electrostatic energy; Δ*E*__vdW_, van der Waals energy; Δ*G*__bind_, binding free energy; Δ*G*__GB_, electrostatic contribution to solvation; Δ*G*__SA_, nonpolar contribution to solvation.

During the simulation, we monitored hydrogen bonding, which is key to determining the binding strength; generally, more hydrogen bonds indicate stronger binding. For example, during the simulation period shown in Figure 5E(d), BAX‐cynecroside formed between 1 and 6 hydrogen bonds. In contrast, the BCL‐2‐cynecroside complex formed 2–3 hydrogen bonds (Figure 5F(d)), and the CDK‐2‐cynecroside complex formed 5–6 hydrogen bonds (Figure 5G(d)). These results show that hydrogen bonds not only persist but also frequently occur throughout the binding process, significantly increasing complex stability.

### 3.5 Pharmacophore Screening

**FIGURE 6:**
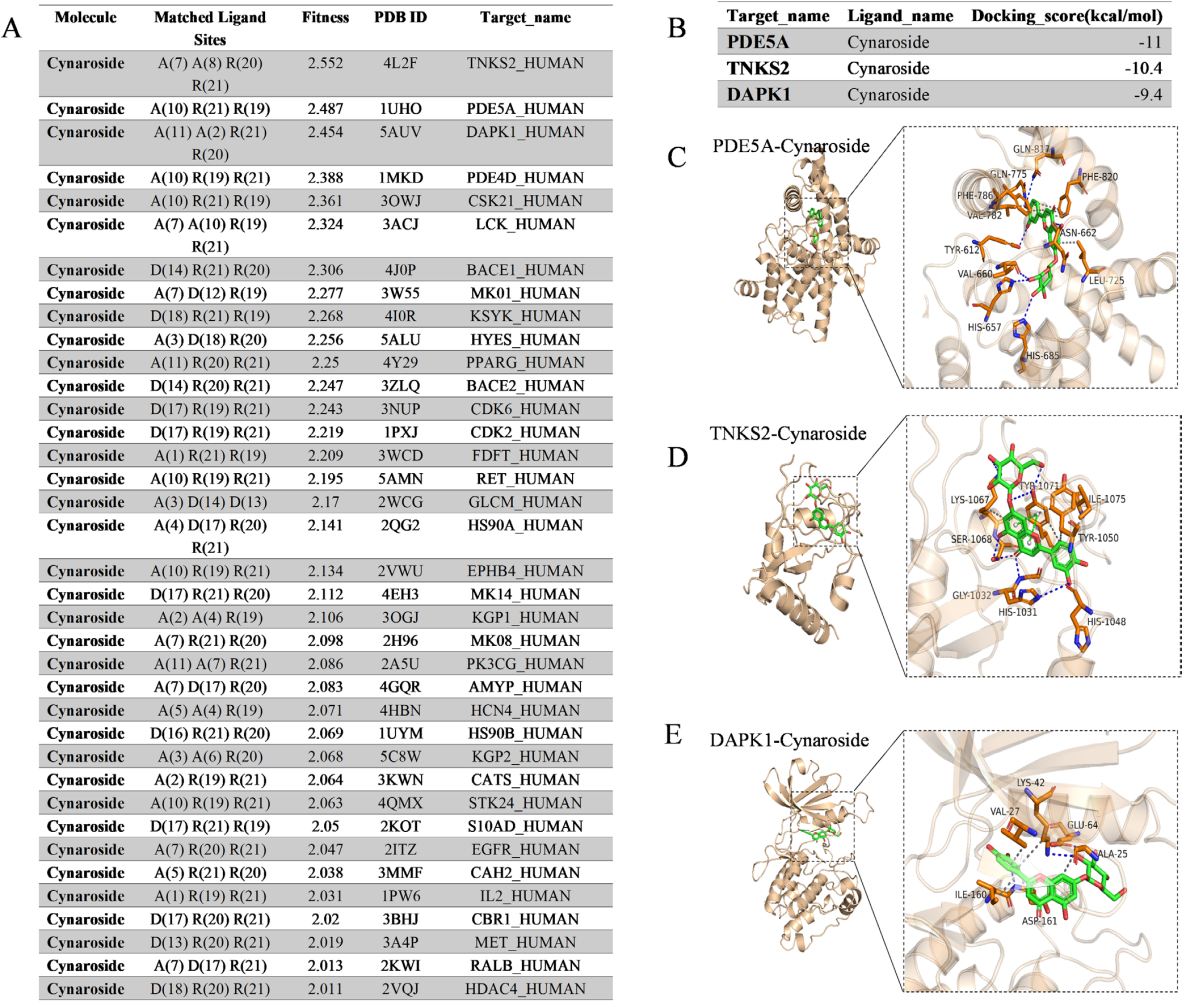
Pharmacophore screening of cynaroside.

Figure 6A displays a carefully curated list of 21 target proteins identified for their high propensity to bind with cynaroside, as deduced from a comprehensive pharmacophore database. The table highlights the fitness scores, where a score of 3 indicates a perfect molecular alignment with the protein's binding site. The figure shows proteins that achieved fitness scores above 2, including TNKS2, PDE5A, and DAPK1, among others, indicating a high potential for interaction with cynaroside. These proteins, selected for their significant fitness values, will be further studied via molecular docking to evaluate the precise binding energies and elucidate the most favorable binding configurations.

Figure 6B shows the docking energies (kcal/mol) of cynaroside for the target proteins PDE5A, TNKS2, and DAPK1, as determined by the Vina docking algorithm. Molecular docking is an essential tool in computational biology, providing insights into the binding energy and conformation between small molecules and target proteins (Nagata 2018). Using Vina software, the docking procedures were conducted, and a docking score below −6.0 kcal/mol generally indicated a strong binding affinity. The reported binding energies for cynaroside with PDE5A, TNKS2, and DAPK1 are all significantly lower than this threshold, confirming the strong interaction of this compound with these proteins. Further investigations will dissect the specifics of these interactions.

Figure 6C displays the complex binding of cynaroside with PDE5A, showing hydrogen and hydrophobic bonds within the protein's helical domain. Cynaroside forms hydrogen bonds with amino acids such as HIS‐657 and ASN‐662 and engages in hydrophobic interactions with acids such as HIS‐657 and ASN‐662, whereas hydrophobic interactions involve VAL‐782 and additional residues. These numerous interactions contribute to the stability of the cynaroside‐PDE5A complex.

Figure 6D shows the binding of cynaroside to TNKS2 in the loop‐rich domain of the protein. It forms hydrogen bonds with GLY‐1032 and HIS‐1048, π‐π stacking with TYR‐1071, and hydrophobic bonds with other residues, indicating that complex interactions stabilize the ophobic interactions with several other residues and illustrating a multifaceted interaction that secures cynaroside to TNKS2.

Figure 6E illustrates the binding of cynaroside‐DAPK1, mainly in the ATP‐binding pocket. Hydrogen bonds with ASP‐161 and GLU‐64 and hydrophobic interactions with LYS‐42 and other residues are noted, highlighting a robust molecular–protein association. The detailed binding dynamics underscore the strong association between small molecules and proteins.

These findings highlight the potential of cynaroside as a biologically active molecule with affinity for proteins such as PDE5A, TNKS2, and DAPK1. Understanding these binding modes enhances the knowledge of its pharmacological potential and suggests applications in the pharmacological potential of cysroside but also opens avenues for its application in drug discovery and therapeutic development.

## 4 Conclusion and Prospects

Cynaroside, a flavonoid compound found widely in plants, has garnered increasing interest for its diverse biological activities. This study successfully extracted cynaroside from peony seed meal via a BTESE/PA membrane, achieving a 90.23% separation rate, confirming the effectiveness of this method for flavonoid extraction. Cynaroside inhibits the proliferation of K562 cells. Cynaroside is a nonchemotherapeutic treatment option for chronic myelogenous leukemia (CML) that potentially reduces side effects such as immune suppression, toxicity, and multidrug resistance. Additionally, Cynaroside triggers cancer cell apoptosis via specific signaling pathways. These findings suggest that cynaroside may both inhibit tumor cell proliferation and promote cell death, effectively reducing the survival rate of cancer cells.

In the clinic, the effects of cynaroside could enhance current cancer treatments or serve as a standalone therapy. In particular, in drug‐resistant CML, cynaroside could complement existing therapies, helping to overcome their limitations. Although in vitro studies have shown that cynaroside has significant anticancer activity, more clinical trials are needed to confirm its safety, efficacy, and optimal dosing. Furthermore, understanding its interactions with other anticancer drugs is crucial for developing precise, personalized treatment strategies. Research on the effects of cynaroside on K562 cell proliferation offers insights into its anticancer mechanisms and paves the way for future clinical applications. Although translating these findings from the laboratory to clinical settings requires extensive validation, the potential of this natural compound for treating neurodegenerative diseases merits further exploration. Future research should aim to fully understand the mechanisms of action of cynaroside, optimize its administration, and assess its efficacy and safety in animal models and humans.

## Author Contributions

**Wen‐Tao Chen:** conceptualization (equal), formal analysis (equal), writing – original draft (equal), writing – review and editing (equal). **Jing Sun:** data curation (equal), software (equal). **Ying‐Yang Zhang:** conceptualization (equal), resources (equal). **Ying Xu:** visualization (equal), writing – original draft (equal), writing – review and editing (equal). **Lei Zhou:** resources (equal), software (equal).

## Ethics Statement

The authors have nothing to report.

## Consent

The authors have nothing to report.

## Conflicts of Interest

The authors declare no conflicts of interest.

## Data Availability

The authors have nothing to report.
